# 

**Published:** 1859-12

**Authors:** 


DETROIT, October 15th, 1859.

MESSRS. EDITORS,—*Dear Sirs:*—The fifth Annual Meeting of the Michigan Dental Association will be held in Detroit, on Tuesday, the 10th day of January next, at 7 o'clock, P. M.

The Committee appointed to select subjects for discussion, report the following:

Difficult Dentition; the effect of Diseased Teeth and Gums on the general health of the system; the proper treatment of Diseased Gums; and the best method of arresting decay in Teeth.

The Committee would intimate that each member of the Association write out what he wishes to say on the above named subjects, to be placed on the records of the Association. If each member would act on the above suggestion, it would make our meeting much more interesting and profitable to the profession.

As there are many new improvements or auxiliaries being introduced to the profession, these meetings can not but be of much interest to every member of the Association, and a large attendance is expected.

Respectfully yours, L. C. WHITING, Sec'y.

### INDIANA STATE DENTAL ASSOCIATION.

*Report of Committee on the order of business for the meeting to be held at Indianapolis, the first Tuesday in Jan.*, 1860.

1st. Reading report of last meeting.

2d. Treasurer's report.

3d. Report of Committees.

4th. Election of Officers.

5th. Installation of Officers.

6th. Appointing Committee.

### DISCUSSION.

*Prop.* 1st. Should Pivot Teeth be inserted? If so, under what circumstances and in what manner?

2d. Treatment of inflamed Dentine.

3d. Treatment of Dental Periostitis and Alveolar abscess.

4th. Mechanical Dentistry, including all the various modes of constructing Artificial Dentines.

5th. Filling Teeth.

6th. Professional intercourse.

7th. Unfinished and Miscellaneous business.

The Committee would respectfully urge the profession throughout the State to attend this meeting, as matters of considerable moment to all practising Dentists will be discussed, as for example, the establishing of a uniform *fee bill*, etc., etc.

J. P. Ulery,<br>

A. M. Moore,<br>

W. R. Webster, } *Committee.*

### Editorial.

### ANOTHER LOCAL SOCIETY.

The dentists of Mad River Valley and vicinity, have organized a society to meet quarterly on adjournment. It held its first regular meeting in Springfield, November 17th. Nine members were present, three essays were read, and a free exchange of professional opinion took place. The next meeting will be in Xenia, on the afternoon and evening of January 5th, 1860. We would be happy to see a full attendance of the profession in the vicinity.

W.

### SANDER'S COMPOUND.

Those who attended the Convention at Niagara, and those who read our report of the discussions, recollect that Dr. Forbes introduced to the notice of the Convention a compound for filling teeth,

### THIRTEENTH VOLUME

OF THE

### DENTAL REGISTER OF THE WEST,

EDITED BY

### J. TAFT and GEO. WATT.

**Issued Monthly. - - - $3 a year in Advance.**

The Volume commences in July. CONTRIBUTIONS to the pages of the Register respectfully solicited.

Exchanges and Correspondents will please address "Dental Register," or J. TAFT, Cincinnati, O; Business communications to JNO. T. TOLAND.

The Register being issued Monthly is always fresh, and in advance of all others, therefore indispensable to the practitioner who desires to keep posted up with the new inventions and improvements which are daily developed.

Neither expense nor effort will be spared to give value and variety to its contents. Articles will be illustrated with well executed engravings, whenever they will add to the interest or assist in explanation.

Its extensive circulation and frequency of issue makes it the BEST DENTAL ADVERTISING MEDIUM in the United States.

### TERMS OF ADVERTISING.

| | One Year. | 6 Mo. | 3 Mo. | 1 Insertion. |
|---|---|---|---|---|
| One Page, | $50 | $30 | $20 | $15 |
| Half | 30 | 20 | 15 | 10 |
| Quarter | 20 | 15 | 10 | 7 |
| One-Eighth | 15 | 10 | 7 | 5 |

**JNO. T. TOLAND, Pub. and Proprietor,**

38 *West Fourth Street, Cincinnati, Ohio.*

